# Pretargeted Imaging beyond the Blood–Brain Barrier—Utopia or Feasible?

**DOI:** 10.3390/ph15101191

**Published:** 2022-09-27

**Authors:** Sara Lopes van den Broek, Vladimir Shalgunov, Rocío García Vázquez, Natalie Beschorner, Natasha S. R. Bidesi, Maiken Nedergaard, Gitte M. Knudsen, Dag Sehlin, Stina Syvänen, Matthias M. Herth

**Affiliations:** 1 Department of Drug Design and Pharmacology, Faculty of Health and Medical Sciences, University of Copenhagen, Universitetsparken 2, 2100 Copenhagen, Denmark; 2 Center for Translational Neuromedicine, University of Copenhagen, Blegdamsvej 3B, 2200 Copenhagen, Denmark; 3 Neurobiology Research Unit, Rigshospitalet Copenhagen University Hospital, Blegdamsvej 9, 2100 Copenhagen, Denmark; 4 Department of Clinical Medicine, University of Copenhagen, 2200 Copenhagen, Denmark; 5 Rudbeck Laboratory, Department of Public Health and Caring Sciences, University of Uppsala, Dag Hammarskjölds väg 20, 75185 Uppsala, Sweden; 6 Department of Clinical Physiology, Nuclear Medicine & PET, Rigshospitalet Copenhagen University Hospital, Blegdamsvej 9, 2100 Copenhagen, Denmark

**Keywords:** pretargeting, *trans*-cyclooctene, antibody, CNS, brain, PET

## Abstract

Pretargeting is a promising nuclear imaging technique that allows for the usage of antibodies (Abs) with enhanced imaging contrast and reduced patient radiation burden. It is based on bioorthogonal chemistry with the tetrazine ligation—a reaction between *trans*-cyclooctenes (TCOs) and tetrazines (Tzs)—currently being the most popular reaction due to its high selectivity and reactivity. As Abs can be designed to bind specifically to currently ‘undruggable’ targets such as protein isoforms or oligomers, which play a crucial role in neurodegenerative diseases, pretargeted imaging beyond the BBB is highly sought after, but has not been achieved yet. A challenge in this respect is that large molecules such as Abs show poor brain uptake. Uptake can be increased by receptor mediated transcytosis; however, it is largely unknown if the achieved brain concentrations are sufficient for pretargeted imaging. In this study, we investigated whether the required concentrations are feasible to reach. As a model Ab, we used the bispecific anti-amyloid beta (Aβ) anti-transferrin receptor (TfR) Ab 3D6scFv8D3 and conjugated it to a different amount of TCOs per Ab and tested different concentrations in vitro. With this model in hand, we estimated the minimum required TCO concentration to achieve a suitable contrast between the high and low binding regions. The estimation was carried out using pretargeted autoradiography on brain sections of an Alzheimer’s disease mouse model. Biodistribution studies in wild-type (WT) mice were used to correlate how different TCO/Ab ratios alter the brain uptake. Pretargeted autoradiography showed that increasing the number of TCOs as well as increasing the TCO-Ab concentration increased the imaging contrast. A minimum brain concentration of TCOs for pretargeting purposes was determined to be 10.7 pmol/g in vitro. Biodistribution studies in WT mice showed a brain uptake of 1.1% ID/g using TCO-3D6scFv8D3 with 6.8 TCO/Ab. According to our estimations using the optimal parameters, pretargeted imaging beyond the BBB is not a utopia. Necessary brain TCO concentrations can be reached and are in the same order of magnitude as required to achieve sufficient contrast. This work gives a first estimate that pretargeted imaging is indeed possible with antibodies. This could allow the imaging of currently ‘undruggable’ targets and therefore be crucial to monitor (e.g., therapies for intractable neurodegenerative diseases).

## 1. Introduction

Positron emission tomography (PET) is a non-invasive nuclear molecular imaging technique that allows for the quantification and visualization of biological processes in vivo with high sensitivity, resolution, and relatively low patient radiation burden [1,2,3,4,5]. PET relies on the administration of radioligands and is commonly used for diagnosis, drug monitoring, or to gain insights into a certain disease pathology [3,4,6,7,8]. The development of new PET radioligands is essential to develop therapies for diseases with respect to the quantification of a drug’s target engagement or to monitor treatment success [9,10,11]. Antibodies (Abs) have high target specificity, selectivity, and typically low non-displaceable binding, making them highly suitable for nuclear PET imaging [12,13,14,15,16].

**Figure 1:**
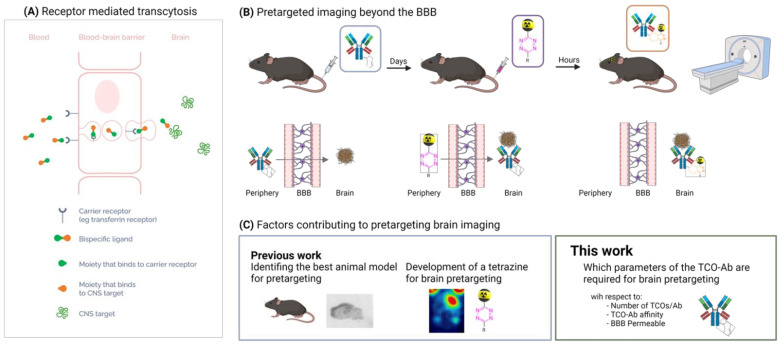
(**A**) An illustration of receptor-mediated transcytosis (RTM). A bispecific ligand binds to its carrier protein and is transported into the brain by transcytosis. (**B**) A schematic overview of pretargeted imaging beyond the BBB. The TCO-Ab is injected first and accumulated at its target within the brain. In a second step, a radiolabeled Tz is administered. This Tz crosses the BBB and reacts with the TCO-Ab. This ligation results in a radiolabeled Ab that can be imaged. (**C**) An overview of the factors contributing to pretargeting in the brain. Previous work describes the identification of animal models and BBB-permeable Tzs. In this work, parameters regarding BBB-permeable TCO-Abs were investigated.

Particularly for brain diseases, the need for PET radioligands is high [17,18,19]. Neurodegenerative disorders are the second most common cause of death, and the number of affected individuals is rapidly growing due to aging societies [20,21,22,23,24]. Developing radioligands for brain targets, however, is not easy and in contrast to tracers imaging targets outside of the brain, the development of central nervous system (CNS) PET tracers is complicated as these tracers have to cross the blood–brain barrier (BBB) [21,25]. This protective barrier prevents toxins from entering and damaging the brain, but also hampers the uptake of pharmacological agents, especially large molecules such as Abs [26,27,28,29,30]. Monoclonal Abs have been revolutionizing PET imaging within oncology, but the application within CNS remains limited due to their poor BBB penetration [12,13]. In order to increase the brain uptake, several mechanisms for facilitated brain delivery are exploited [20,26,31,32,33,34]. The most commonly exploited strategy is receptor-mediated transcytosis (RMT), which involves the conjugation of an Ab to a transporting moiety. This moiety binds to a transporter (carrier) and consequently delivers the construct into the brain (Figure 1A) [35,36,37]. The transferrin receptor (TfR) is the most commonly used transporter thus far [38,39,40,41].

Abs often have slow pharmacokinetics and long circulation times and therefore it can take several days before an Ab has accumulated at its target and is excreted from the rest of the body. As such, Abs have to be labeled with long-lived radioisotopes (e.g., zirconium-89 or iodine-124). These radionuclides typically result in relatively high patient radiation burden [42,43]. A strategy to circumvent the use of long-lived radioisotopes is pretargeting. In this strategy, a two-step approach is used where a non-radioactive, tagged Ab is administered first and allowed to accumulate at its target and at the same time eliminated from the blood. In a second step that is usually carried out a couple of days later, a radiolabeled molecule—often a small molecule with rapid clearance from blood—is administered. Subsequently, this radiolabeled molecule reacts rapidly and selectively in vivo with the tagged Ab (Figure 1B) [44,45]. Thus, pretargeting allows for the use of short-lived isotopes such as fluorine-18 [46]. The most attractive reaction for pretargeting is tetrazine ligation [45]. This ligation is highly selective and ultra-fast and is based on the reaction between a tetrazine (Tz) and a *trans*-cyclooctene (TCO). Successful pretargeting in the periphery was shown by Rossin et al. for the first time using a ^111^In-labeled Tz in combination with a TCO-modified CC49 mAb [47]. The in vivo performance of the Tz ligation depends on the properties of the Tz as well as on those of the TCO. Suitable Tzs should be stable, highly reactive, clear rapidly from the blood stream, and easy to radiolabel. We have recently developed robust methods to radiolabel highly reactive Tzs with fluorine-18 as it is the most relevant radionuclide for clinical PET studies [48,49,50,51]. For brain pretargeted imaging, the Tz must also enter the brain. Such Tzs have also been developed by us [52]. Usually, TCOs are conjugated to the Ab as this increases the TCO stability by reducing the isomerization rate to its unreactive cis-cyclooctene (CCO) form [45,53,54]. For pretargeting, the TCO concentration per Ab should be as high as possible without significantly interfering with the properties of the Ab. A higher TCO concentration increases the chances of a Tz to react in vivo with the Ab when administered in a second step. Finding the right balance between TCO-loading, stability, and reactivity is therefore crucial when preparing TCO-Ab conjugates [45,53,54].

In this study, we investigated whether the TCO-concentrations required for pretargeted imaging are feasible to reach within the brain using a BBB shuttle bispecific Ab. For this purpose, we modified the monospecific anti-amyloid beta (Aβ) Ab 3D6 and bispecific anti-Aβ anti-transferrin receptor (TfR) Ab 3D6scFv8D3 [55,56] with different concentrations of TCOs and quantified the minimum TCO concentration to achieve a suitable contrast between the high and low binding regions. The contrast was determined using pretargeted autoradiography on brain sections of an Alzheimer´s disease mouse model. Additionally, biodistribution studies in wild-type mice were carried out to correlate how different TCO/Ab ratios alter the brain pharmacokinetics of our bispecific Ab. Figure 1C displays the objectives of this study.

## 2. Results and Discussion

### 2.1. Theoretical Considerations to Determine the Minimum Required TCO Concentration

As with any imaging approach, a reasonable contrast between target-rich and target-poor (high and low binding) regions is needed. The contrast is dependent on the tracer itself—specific binding vs. non-displaceable binding—as well as on the maximum available concentration of the target. In pretargeted approaches, TCOs are the target of interest and as such, it is interesting to estimate the minimum TCO concentration required to distinguish the high from low binding regions. To estimate this minimum concentration, we assumed that the in vitro contrast determined by autoradiographic studies can be extrapolated to the in vivo situation when a Tz is used that is known to result in clear in vitro and in vivo contrast. ^111^In-labeled 1,4,7,10-tetraazacyclododecane-1,4,7,10-tetraacetic acid (DOTA)-PEG_11_-tetrazine (^111^In-Tz) has been successfully used for in vivo pretargeted imaging and has low non-specific binding due to its high hydrophilicity [47,57]. Therefore, we chose ^111^In-Tz as a model Tz for this study. We decided to set the threshold for the high/low binding region ratio to be a minimum of 2. This value appeared reasonable as such a ratio can be visually distinguished and is representative of the performance of PET tracers used to diagnose neurodegenerative diseases in the clinic such as the beta-amyloid imaging tracer [^11^C]PIB [58].

In the in vitro pretargeted autoradiography setup, the minimum TCO concentration (TCO_min_) required to distinguish between high and low binding regions can be expressed through the following parameters: the minimum amount of Abs ($n_{min}^{Ab}$) applied to the tissue slice that provides a sufficient imaging contrast (high/low binding region ratio ≥2), the percentage of applied Abs that bind to the tissue ($\eta_{b}$), the number of reactive TCO moieties per Ab ($N_{TCO}$), and the volume of the tissue sample that was tested (v_tiss_). The relationship between these parameters is shown in Equation (1). Once the parameters in the right part of the equation are determined experimentally, the TCO_min_ can be calculated.

(1) $$TCO_{min}=\frac{n_{min}^{Ab}\times \eta_{b}\times N_{TCO}}{v_{tiss}}$$

where *TCO_min_* = the minimum required TCO-concentration per gram brain tissue (nmol/g); $n_{min}^{mAb}$ = the minimum amount of TCO-Abs that provides an in vitro image; $\eta_{b}$ = the percentage of Abs that become bound to the tissue (%); *N_TCO_* = the number of reactive TCOs per mAb; *v_tiss_* = the tissue volume (μL).

*TCO_min_* can also be converted into the Ab concentration (*Ab_min_*) by dividing it by the TCO loading, as shown in Equation (2).

(2) $$Ab_{min}=\frac{TCO_{min}}{N_{TCO}}$$

where *Ab_min_* = the minimum required Ab concentration per gram brain tissue (nmol/g); *TCO_min_* = the minimum required TCO-concentration per gram brain tissue (nmol/g); *N_TCO_* = the number of reactive TCOs per mAb.

*Ab_min_*, determined from the in vitro experiments, can be compared with the Ab concentration that can be trafficked over the BBB in in vivo experiments (*Ab_traffic_*). We expect pretargeted imaging over the BBB to be possible when *Ab_min_* is the minimum in the same order of magnitude as *Ab_traffic_* (Equation (3)).

Success criteria that the brain pretargeting works:

(3) $$Ab_{traffic}\geq Ab_{min}$$

*Ab_traffic_* = the Ab concentration that can be trafficked over the BBB (nmol/g); *Ab_min_* = the minimum required Ab concentration per gram brain tissue (nmol/g).

### 2.2. Antibody Modifications and TCO Quantification (N_TCO_)

**Figure 2:**
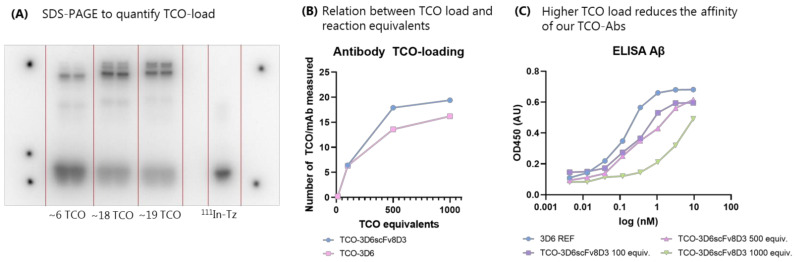
(**A**) SDS-PAGE with ^111^In-Tz and TCO-Abs to quantify the TCO load. Activity at the bottom corresponds to the ^111^In-Tz and the activity bands at the top to the radiolabeled Ab. The difference in radioactivity was used for the TCO quantification (Supplementary Materials). The lanes on the left correspond to ~6 TCO/Ab, and the middle lanes to ~18 and ~19 TCO/Ab TCO/Ab. The lane on the right is the ^111^In-Tz without Ab (control). (**B**) Different TCO-loading can be reached using different equivalents of TCO. The saturation of TCO-loading was reached around 20 TCOs. (**C**) ELISA for Aβ with Ab 3D6 and TCO-3D6scFv8D3. Affinity was reduced with an increased amount of TCOs.

TCO-PEG_4_-NHS was conjugated in a non-site-selective manner to the lysine residues of the Abs. Due to the high prevalence of lysines on proteins, this strategy allowed us to reach a wide range of TCOs conjugated to the Ab [43,59]. Different equiv. of TCOs—ranging from 10–1000 equiv.—were added to 3D6 and mAb3D6scFv8D3. The number of reactive TCOs was determined by titration. TCO-Abs was incubated with an excess of ^111^In-Tz and then applied to an SDS-PAGE gel. Exposure of these SDS-PAGE gels to phosphor screens allowed for the quantification of the TCO moieties per Ab (Figure 2A) (Supplementary Section S2). A maximum of approx. 19 TCOs/Ab could be reached (Figure 2B). Interestingly, 500 equiv. of TCO-PEG_4_-NHS resulted in a similar TCO-loading compared to 1000 equiv.

However, the ELISA data showed a significant reduction in affinity to both Aβ and TfR when 1000 equiv. was used, and therefore this TCO-Ab was excluded for further examination (Figure 2C and Supplementary Section S9). TCO-3D6scFv8D3 up until 500 equiv (giving approx. 15 TCO/Ab) was used for further evaluation. The affinity reduction can be explained by the non-site-specific conjugation to the lysine residues [9]. Lysine residues were also present in the complementarity-determining region (CDR), meaning that alterations to this binding domain can occur when adding TCOs. As previously mentioned, higher TCO-load reduced the affinity of the Ab construct toward TfR and therefore, we also determined the influence of the TCO load on the brain accumulation of this construct (biodistribution section).

### 2.3. Pretargeted Autoradiography to Determine the Minimum Amount of Abs ($n_{min}^{mAb}$) Required for Sufficient Imaging Contrast

**Figure 3:**
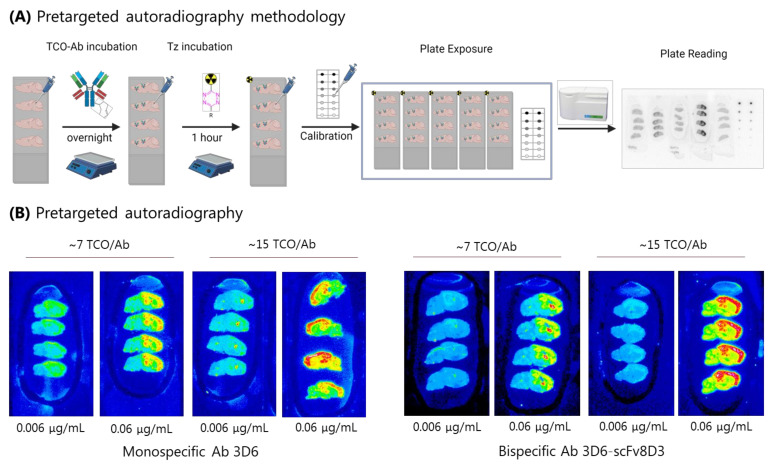
(**A**) The pretargeted autoradiography workflow. TCO-Abs was applied first and incubated overnight. Then, the radiolabeled Tz was applied. Slides were washed and a calibration curve was added before slide exposure to the phosphor plates. Phosphor plates were read-out to provide the autoradiography images. (**B**) Examples of the autoradiography results with TCO-3D6 and TCO-3D5scFv8D3. Increasing TCO-loading and TCO-Ab concentration increased the imaging contrast. Similar results were observed for TCO-3D6 and TCO-3D5scFv8D3.

Autoradiography is an imaging technique that allows for the visualization of radioligands on tissues of interest and thus to determine the imaging contrast of novel CNS radioligands, for example, on brain sections [60,61]. In order to determine the imaging contrast that is feasible to reach with pretargeting, we performed pretargeted autoradiography experiments according to our previously published pretargeted autoradiography methodology [52]. Brain sections from Tg-ArcSwe mice were used for these studies. Tg-ArcSwe mice possess high Aβ pathology in the cortex (cor, high binding region), low pathology in the cerebellum (cer, low binding region), and have successfully been used for PET Aβ imaging with [^124^I]3D6scFv8D3 [62]. Pretargeted autoradiography is a two-step imaging approach and simulates in vivo pretargeting as close as possible (Figure 3A). Brain sections were first incubated overnight with TCO-3D6 or TCO-3D6scFv8D3. The next day, the excess of mAbs was washed away. In the second step, the brain sections were incubated with ^111^In-Tz for one hour. This timeframe was sufficient to achieve complete ligation of the available TCOs as ^111^In-Tz is a bispyridyl tetrazine with an expected reaction timeframe within minutes [49]. Afterward, the excess ^111^In-Tz was washed away and the sections were quantified and analyzed using the uptake values in the cortex and in cerebellum.

This methodology allowed for the determination of the imaging contrast using TCO-Abs with different TCO loadings at different concentrations. Images with TCO-3D6 and TCO-3D6scFv8D3 revealed that an increased imaging contrast was achieved by increasing the TCO-Ab concentration as well as by increasing the number of TCOs on the Ab (Figure 3B). An increased imaging contrast of 1.5-fold could be observed when increasing the absolute TCO number per Ab from 7 to 15 for both Abs. To assess the dependence of the imaging contrast on Ab concentration, TCO-3D6 and TCO-3D6scFv8D3 were incubated at different concentrations, ranging from 0 to 0.20 μg/mL (Supplementary Section S10). The cor/cer ratios were determined. A ratio >2 was observed for both TCO-Abs using 0.06 μg/mL. At this concentration, a clear distinct uptake in the high and low binding regions was visually evident. A cor/cer ratio of approx. 1.3 was determined for 0.02 μg/mL. Consequently, only the 0.06 μg/mL concentration fulfilled our contrast requirement (i.e., a ratio of >2 with respect to the high-to-low binding region uptake). A total of 0.06 μg Ab translated to a $n_{min}^{mAb}$ of 0.17 pmol TCO-3D6scFv8D3.

### 2.4. Determination of TCO-Abs ($\eta_{b}$) Bound to Tissue and Tissue Volume (v_tiss_)

The percentage of TCO-Ab bound to brain sections ($\eta_{b}$) was determined by gamma counting. TCO-Ab conjugate with 3.6 TCOs/Ab was ligated with ^111^In-Tz. The resulting ^111^In-labeled Ab was applied to the brain sections. Unbound ^111^In-labeled Ab was washed off and the brain sections were wiped off the glass plate and measured by a gamma counter. A $\eta_{b}$ of 7% was determined.

Brain sections of 20 μm were used with four sections per slide. Based on the average brain sizes of the C57BL/6J mice, a total tissue volume (v_tiss_) of approx. 4 μL was estimated per slide [63,64].

### 2.5. Minimum Required TCO Concentration In Vitro

Equation (1) was used to calculate the *TCO_min_*. $n_{min}^{mAb}$, $\eta_{b}$, *N_TCO_*, and *v_tiss_* were determined/estimated as described in previous sections. A *TCO_min_* of 10.7 pmol/g of TCOs was determined. This amount is needed to reach the minimum high binding/low binding ratio of 2.

$$TCO_{min}=\frac{n_{min}^{mAb}\times \eta_{b}\times N_{TCO}}{v_{tiss}}=\frac{0.17\;pmol\times 7\%\times 3.6}{4\;uL}=10.7\;\mathrm{pmol}/g$$

### 2.6. Biodistribution TCO-3D6scFv8D3–Determination of Ab_traffic_

**Figure 4:**
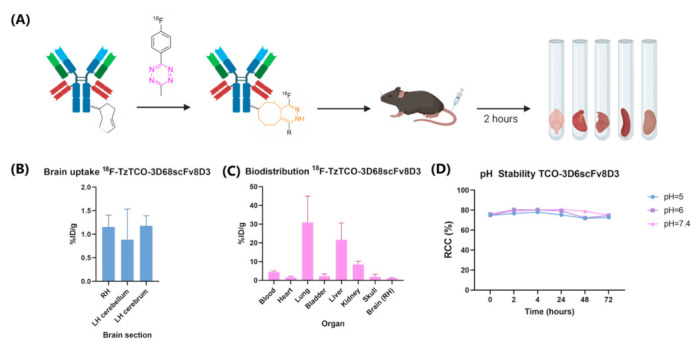
(**A**) Experimental setup biodistribution study. TCO-3D6scFv8D3 was reacted in vitro to ^18^F-tetrazine to obtain the ligated product. ^18^F-3D6scFv8D3 was injected into the WT mice. After two hours, the mice were euthanized, and the organs were collected and measured by gamma counting. (**B**) Brain uptake measured by gamma counting. RH = Right hemisphere. LH = Left hemisphere (**C**) Biodistribution of ^18^F-3D6scFv8D3. (**D**) pH stability of TCO-3D6scFv8D3 showing a stability at over three days at pH 5, 6, and 7.4. TCO-3D6scFv8D3 was incubated with an excess of ^111^In-Tz and the TCO-reactivity was monitored by evaluating Tz ligation using radio-HPLC.

To estimate which Ab concentration of TCO-3D6scFv8D3 can enter the brain in vivo (*Ab_traffic_*), a biodistribution study in wild-type (WT) mice was conducted. We decided to use TCO-Ab conjugates containing approx. 7 TCO/Ab, as this ratio did not result in a significant decrease in the TfR affinity compared to the unmodified Ab (Supplementary Materials). TCO-Abs containing ~7 TCO/Ab has also been successfully used for tumor pretargeting and as such, we believe that this TCO loading was a reasonable starting point [47]. TCO-3D6scFv8D3 containing 6.8 TCO/Ab was labeled with an ^18^F-labeled Tz (Figure 4A). An excess of TCO-Ab (1:9 Tz/TCO ratio) was used to avoid an additional purification step. Consequently, the protein losses and changes in the protein concentration could be minimized, and therefore, the TCO-Ab concentration in the solution was precisely known during the experiment. The WT mice with an average weight of 25 g were injected with 50 μg of Ab, corresponding to a dose of approx. 2 mg/kg. After two hours, the animals were euthanized, a total of 16 organs were collected, and the radioactivity amount was quantified. The percentage injected dose per gram tissue (%ID/g) was calculated (Figure 4B). A brain uptake of 1.1% ID/g was detected, which agreed with the published data for the unmodified 3D6scFv8D3 [65]. Among the peripheral organs, the uptake was mainly observed in the lungs and the liver (Figure 4C). An overview of the uptake in all organs can be found in Supplementary Section S11.

### 2.7. Is Pretargeting Feasible? Ab_min_ vs. Ab_traffic_

We estimated that *Ab_traffic_* needs to be in the same order of magnitude or higher than *Ab_min_* to enable sufficient imaging contrast in pretargeted brain imaging (Equation (3)). *Ab_min_* can be calculated according to Equation (2). *TCO_min_* was determined to be 10.7 pmol/g and the TCO loading per Ab used determined that *Ab_traffic_* was 6.8. As this bispecific Ab can enter the brain with an ID/g of 1.1% and shows an affinity toward Aβ that is comparable to the unmodified Ab version (Figure 2C), we believe that this TCO amount can be applied for in vivo pretargeted imaging. Consequently, *Ab_min_* was determined to be 1.6 pmol/g.

$$Ab_{min}=\frac{TCO_{min}}{N_{TCO}}=\frac{10.7\;pmol/g}{6.8}=1.6\;\mathrm{pmol}/g$$

An in vivo brain uptake of 1.1% ID/g using a dosage of 50 μg resulted in a total of 2.6 pmol/g TCO-Ab (*Ab_traffic_*) that was able to enter the brain. This value was higher than the calculated theoretical value, and consequently, the amount of required *Ab_min_* and measured Ab_traffic_ were in the same order of magnitude. These estimations indicate that pretargeted imaging beyond the BBB is feasible using the described setup (Table 1).

### 2.8. pH Stability of TCOs Conjugated to 3D6scFv8D3

Substances that are transported via RMT into the brain were exposed to different pH values ranging from approx. 5 to 7.5 [66]. Since TCOs are acid-sensitive, we investigated the stability of TCOs conjugated to 3D6scFv8D3 at different pH values [67]. At all of the tested pH values, TCOs were stable and reactive to ^111^In-Tz (Figure 4D).

## 3. Materials and Methods

### 3.1. Materials

Unless otherwise stated, all reagents and solvents were purchased from commercial suppliers and used without further purification. All of the water used was ultrapure (>18.2 MΩ cm^−1^). Other solvents were of analytical or HPLC grade and used as received.

### 3.2. General Information

Radiochemistry was performed at the Department of Clinical Physiology, Nuclear Medicine and PET, Rigshospitalet, Denmark. [^18^F]Fluoride was produced by the (p,n) reaction in a cyclotron (60 mikroA CTI Siemens or 40 mikroA Scanditronix) by irradiating [^18^O]H_2_O with a proton beam of 11 MeV (CTI siemens) or 16 MeV (Scanditronix). [^111^In]InCl_3_ was purchased from Curium. Automated syntheses were performed in a Scansys Laboratorieteknik synthesis module housed in a hot cell. The analytical-HPLC system consists of a 170U UVD detector, a Scansys radiodetector, and a Dionex system connected to a P680A pump. The system was run by Chromeleon software.

The radiochemical conversion (RCC) was determined by analyzing an aliquot of the crude reaction mixture by radio-HPLC analysis, integrating the radioactive peaks of the chromatogram. Radiolabeled products were characterized by associating the UV-HPLC traces of the reference compounds with the radio-HPLC chromatogram of the radiotracer. The radiochemical yield (RCY) was determined using the initial activity at the beginning of the synthesis and that of the formulated product at the end of the synthesis (E.O.S.) and corrected for decay (d.c.). The molar activity (Am) was determined by integrating the area of the HPLC-UV absorbance peak of the radiolabeled product in the HPLC chromatogram. The radiochemical purity (RCP), radiochemical yield (RCY), and molar activity (Am) values are given as the mean values.

Radiochemical conversion (RCC) of all radiolabeled compounds was determined by analyzing a labeled aliquot of the reaction mixture by radio-HPLC and analyzed by integrating the radioactive peaks from the reaction solution. The products were characterized by comparing the radio-HPLC trace of the reaction mixtures with the HPLC UV traces of the authentic ^19^F-reference samples, respectively. The radiochemical yield (RCY) was determined using the activity of [^18^F]fluoride received from the cyclotron at the beginning of the synthesis and that of the formulated product at the end of the synthesis. The decomposition was corrected and decay corrected (d.c.). The molar activity (Am) was determined by integrating the area of the UV absorbance peak corresponding to the radiolabeled product on the HPLC chromatogram. This area was converted into a molar mass by comparison with an average of integrated areas (triplicates) of a known concentration for the corresponding reference compounds.

### 3.3. Radiolabeling of Tz [^18^F]1

Reference compound **1** was prepared according to the procedure previously reported.^4^ [^18^F]**1** was radiolabeled from the precursor 3-(4-trimethyltin)-6-methyl-1,2,4,5-tetrazine (**1b**) as previously described by García-Vázquez et al., Scheme S1). Tz [^18^F]**1** was afforded in a radiochemical yield (d.c.) of 25 ± 7%, a radiochemical purity (RCP) ≥98% and a specific activity (A_m_) of 190 ± 10 GBq/μmol (d.c) (*n* = 3). A typical activity yield was 2.5–3 GBq starting from ~12 GBq [^18^F]fluoride, and the total duration of the synthesis was 1 h.

### 3.4. Radiolabeling of TCO-3D6scFv8D3 with Tz [^18^F]1

To a 1.5 mL Eppendorf vial was added TCO-3D6scFv8D3 containing 6.8 TCO/Ab (700 μL, 2.47 nmol protein, 16.77 nmol TCO), and Tz [^18^F]**1** (Am (d.c.) = 183.2 GBq/mL, RCP = 98%, carrier Tz = 2.26 nmol/mL, 0.43 ug/mL, 115 MBq, 519.02 μL, 1.17 nmol, 0.07 TCO/Tz eq.). The mixture was shaken at 600 rpm for 30 min at 37 °C, after which the full conversion of ^18^F-Tz was observed by radio-HPLC, yielding the [^18^F]3D6scFv8D3 construct with an radiochemical purity (RCP) of >96%, as determined by radio-TLC (silica gel, 80:20 ratio ethyl acetate/n-heptane). The radiotracer was formulated in PBS to obtain a final protein concentration of 416 μL/mL (approximately 1.5 MBq/mL at the time of tracer delivery) and used for the animal experiments (Section S7).

### 3.5. Radiolabeling of Tz [^111^In]2

^111^In-labeled Tz **2** was used for the titration of the TCO-modified antibodies (Section S2) and for the preparation of [^111^In]3D6scFv8D3 (next section). The labeling was performed as previously described (Scheme S3) [52]. 1,4,7,10-Tetraazacyclododecane-1,4,7,10-tetraacetic acid (DOTA)-PEG_11_-tetrazine (**2**) was dissolved (2 mg/mL) in metal-free water and stored at −80 °C before use. An aliquot of 50–100 μL (10–30 MBq) of [^111^In]indium chloride in 0.05 M HCl was combined with 2 μL DOTA-PEG_11_-tetrazine and 1 M NH4OAc buffer (pH 5.5) at a volume ratio of 1:10 was added. The mixture was shaken at 600 rpm for 5 min at 60 °C in an Eppendorf ThermoMixer C. Then, 10 mM diethylenetriamine-pentaacetic acid DTPA (volume ratio 1:10) and 2 μL 10 mg/mL gentisic acid in saline was added and the solution was shaken for an additional 5 min at 60 °C in an Eppendorf ThermoMixer C. Typically, a quantitative labeling yield and a radiochemical purity (RCP) greater than 95% were obtained with this method, as confirmed by radio-HPLC (Aeris Peptide C18-XB 3.6 µm 150 × 4.6 mm column. Solvent A—0.1% TFA in water, solvent B—0.1% TFA in acetonitrile. HPLC elution method: 0–1 min–5% B, 1–8 min-gradient from 5% B to 75% B, 8–9 min–75% B, 9–9.5 min-back to 5% B, 9.5–10 min–5% B; flow rate 1.5 mL/min).

### 3.6. Radiolabeling of TCO-3D6scFv8D3 with Tz [^111^In]2

To a 1.5 mL Eppendorf vial was added TCO-3D6scFv8D3 containing 3.4 TCO/Ab (80 μL, 0.28 nmol TCO) and Tz [^111^In]**2** (RCP = 99%, 0.14 nmol, 0.5 Tz/TCO eq). The mixture was shaken at 600 rpm for 60 min at 37 °C, after which full conversion of the ^18^F-Tz was observed by radio-HPLC, yielding the [^111^In]3D6scFv8D3 construct with a radiochemical purity (RCP) of >96%, as determined by radio-HLPC (Aeris™ 3.6 µm Widepore C4 200 Å, LC Column 150 × 4.6 mm, Solvent A = 0.1% TFA in water, solvent B = 0.1% TFA in acetonitrile. HPLC elution method: 0–4.5 min–25% B, 4.5–8 min-gradient from 25% B to 90% B, 8–10.5 min–90% B, 10–12.5 min-back to 25% B, 12.5–15 min–25% B; flow rate 1.5 mL/min). [^111^In]3D6scFv8D3 was used for the in vitro experiments described in Section S6.

## 4. Pretargeted Autoradiography

Pretargeted autoradiography was performed according to the previously published procedure [52]. A schematic overview is provided in Section S5.

Frozen brains of old (~18 months of age; 25–40 g body weight) male C57BL/6 mice and the tg-ArcSwe model harboring the Arctic (AβPP E693G) and Swedish (AβPP KM670/671NL) mutations (provided by Uppsala University) were cut into halves along the sagittal symmetry plane. Each half was mounted, lateral side up, on the cryostat sample holder pretreated with the Tissue-Tek OCT Compound, and fixed by freezing in a cryostat. After fixing, brains were cut at −22 °C into sagittal slices 20 μm thick using a Leica microtome, and the slices were thaw-mounted on Superfrost (70 × 22 mm, Fischer) adhesive slides. Only slices containing both cortex (specific binding) and cerebellar regions (representing nonspecific binding) were used. The slices were allowed to dry and were then put into storage boxes and kept at −80 °C until they were used.

On the day of the experiment, the frozen slides with the mounted brain sections were allowed to come to room temperature for 30 min. Afterward, a PAP pen was applied around the sections and 600 μL 1% BSA in PBS (pH 7.4) with 0.05% Tween 20 was added and incubated at room temperature for 30 min in incubation boxes. Afterward, the BSA solution was removed and 600 μL of PBS (pH 7.4) with 0.05% Tween 20 was applied and incubated at room temperature for 5 min. The preincubation buffer was removed and 600 μL of TCO-3D6 or TCO-3D6scFv8D3 (0.06 μL/mL and 0.006 μL/mL) in PBS (pH 7.4) with 0.05% Tween 20 was applied and incubated overnight at room temperature in a moist incubation box. On the second day, the TCO-mAb solution was removed and the slides were washed in cold PBS (pH 7.4) (3 × 15 min) and cold demineralized water (1 × 30 s). Then, 600 μL 20 nM [^111^In]**2** solution was applied to all sections and incubated for 60 min. Afterward, the radioactive solution was removed and the slides were washed in cold PBS (pH 7.4) (3 × 5 min) and demineralized water (1 × 30 s) and dried by a flow of compressed air. A calibration curve with dilutions of [^111^In]**2**-40 nM, 20 nM, 6.7 nM, 2.2 nM, 0.74 nM, 0.25 nM, and 0.12 nM was prepared. After drying the plates, the slides were exposed on phosphor storage screens for 1–12 h, depending on the amount of radioactivity on the slide. Afterward, the phosphor storage screens were read by a Cyclone Storage Phosphor System (Packard Instruments Co., Meriden, CT, USA). Quantification of the plate readings was conducted with Optiquant software (version 3.00, Packard Instruments Co.) and ImageJ by drawing regions of interest on the cortex and cerebellum manually. Data were processed in Excel and the cortex/cerebellum imaging contrast ratios were calculated.

## 5. Determination of TCO-Ab Bound to Tissue

Slides with brain sections from the tg-ArcSwe mice were prepared as described in Section S6. Frozen brain slides were allowed to acclimatize to room temperature for 30 min. A PAP pen was applied around the sections and 600 μL 1% BSA in PBS (pH 7.4) with 0.05% Tween 20 was added and incubated at room temperature for 30 min in incubation boxes. Afterward, the BSA solution was removed and 600 μL of PBS (pH 7.4) with 0.05% Tween 20 was applied and incubated at room temperature for 5 min. The preincubation buffer was removed and 600 μL of [^111^In]3D6scFv8D3 (0.06 μL/mL and 0.006 μL/mL) in PBS (pH 7.4) with 0.05% Tween 20 was applied and incubated overnight at room temperature in a moist incubation box. [^111^In]3D6scFv8D3 was prepared as described in Section S4. On the second day, the [^111^In]3D6scFv8D3 solution was removed and the slides were washed in cold PBS (pH 7.4) (3 × 5 min) and cold demineralized water (1 × 30 s). After drying the plates, the brain tissue was wiped off with tissue paper and placed in γ-counting tubes and measured by counting with the γ-counter Cobra II Auto-Gamma Counter (Model D5005, Packard BioScience Company). Quantification of the γ-counts was conducted in Excel to determine the TCO-Abs bound to the tissue.

## 6. Ex Vivo Biodistribution of [^18^F]3D6scFv8D3

### 6.1. Animals

Ex vivo distribution experiments were performed with 12-week old male C57BL/6JRj mice purchased from Janvier Labs. Mice were maintained at Panum, AEM Unit 16.2, University of Copenhagen and arrived 4 weeks prior to the experiments for habituation in the new environment. Mice were housed together in individually ventilated cages in a specific pathogen-free, humidity and temperature-controlled facility (12 h light/dark cycle) with free access to water and chow. All procedures were conducted following the European Commission’s Directive 2010/63/EU, FELASA, and ARRIVE guidelines for animal research and were approved by The Danish Council for Animal Ethics (license number 2020-15-0201-00581).

### 6.2. Animal Injection and Ex Vivo Biodistribution Procedure

For the ex vivo biodistribution of [^18^F]3D6scFv8D3, mice (*n* = 3) were anaesthetized with the injectable anesthesia mix of ketamine (100 mg/kg) and dexmedetomidine (0.5 mg/kg) and kept in anesthesia during the whole experiment. To maintain a physiological body temperature, mice were kept on a warming pad. All animals were tested for pain response by toe pinching. 26G venous catheters (26G BD Neoflon™, VWR) were inserted in the lateral tail veins and fixed with tape. A total of 50 μg of ^18^F-3D6scFv8D3 (equaling a volume of 120 μL and between 0.5 and 0.7 MBq per mouse) was injected followed by a saline flush of 80 μL. Two hours after antibody injection, mice were euthanized by an injection of a lethal dose of ketamine/dexmedetomidine (300 mg/kg and 1.5 mg/kg). Blood was collected via cardiac puncture and separated into a plasma and a blood cell fraction using EDTA tubes centrifuged at 4 °C at 2000× *g* for 10 min.

After blood collection, mice were perfused with ice-cold saline and the following tissues were collected: heart, lungs, bladder, liver, stomach, gall bladder, kidneys, bone, muscle, scull, spleen, right brain hemisphere, left cerebellum, and left cerebrum. All tissues were wet-weighed and counted on an automated γ-counter Cobra II Auto-Gamma Counter (Model D5005, Packard BioScience Company). The concentration of radioactivity in the blood and tissues was determined as percentage injected dose per gram (%ID/g). Data were expressed as mean ± SD (*n* = 3).

## 7. TCO Stability in TCO-3D6scFv8D3 at Different pH

TCO-3D6scFv8D3 containing approx. 8 TCOs/Ab in PBS was divided into three aliquots and the pH was altered to give a final pH of 4, 5, and 7.4,k respectively. After 0, 2, 4, 24, 48, and 72 h, an aliquot of each vial was taken and an excess of ^111^In-Tz (1.5 eq) was added. The mixtures were incubated for one hour at 37 °C and measured by radio-HPLC (Aeris™ 3.6 µm Widepore C4 200 Å, LC Column 150 × 4.6 mm, Solvent A = 0.1% TFA in water, solvent B = 0.1% TFA in acetonitrile. HPLC elution method: 0–4.5 min–25% B, 4.5–8 min-gradient from 25% B to 90% B, 8–10.5 min–90% B, 10–12.5 min-back to 25% B, 12.5–15 min–25% B; flow rate 1.5 mL/min). Peaks were integrated to determine the RCC.

## 8. Conclusions

We investigated whether pretargeting beyond the BBB is feasible. Based on our theoretical considerations and experimental data, we believe that it is. The required TCO concentrations can most likely be achieved without altering the current application strategies. However, pretargeting beyond the BBB is not only dependent on the TCO concentration that can be reached within the brain, but also on the ability of the tetrazine to reach its target and be rapidly cleared from the brain tissue and the rest of the body. This is of special importance for Tzs labeled with fluorine-18 with a half-life of approximately 110 min, which we have developed previously [52]. Finally, the imaging contrast of pretargeted imaging is dependent on the efficient clearance of unbound TCO-modified antibody still circulating in the blood. Unbound TCO-Ab has more time to clear from the brain in pretargeted strategies compared to imaging with directly labeled antibodies, and, if needed, clearing agents can be used to reduce unbound TCO-modified Abs circulating in the blood. These agents can be based on tetrazine ligation, where columns loaded with Tzs can be connected to the blood stream to trap the unbound TCO-Ab prior to the administration of the radiolabeled Tz. The imaging contrast was increased 125-fold by removing the circulating antibody in a cancer case [68]. This increase in imaging contrast could be needed to image brain targets with low abundancy. In conclusion, we predict that the TCO concentration within the brain achievable by conventional single-bolus i.v. injection of TCO-Abs is high enough for pretargeting beyond the BBB. Previously, we have developed Tzs that can cross the BBB, can successfully ligate the deposited targets in vivo and is able to image these targets within the brain [52]. In light of this, our next steps are directed toward the study of the possibility of using TCO-3D6scFv8D3 for pretargeted imaging in the Tg-ArcSwe mice in vivo. Additionally, brain uptake with TCO-Abs containing more than 6.8 TCO/Ab could be of interest to test, since this could increase the imaging contrast and lower the required dose. We believe that pretargeting beyond the BBB with antibodies will allow for the imaging of currently ‘undruggable’ targets and would therefore be crucial to monitor the therapies for currently untreatable neurodegenerative diseases.

## Figures and Tables

**Table 1:** A summary of the results. Pretargeting beyond the BBB appears to be feasible as the minimum amount of TCOs required for pretargeted brain imaging can be delivered using the RMT brain delivery of TCO-3D6scFv8D3 (i.e., *Ab_traffic_* ≤ *Ab_min_*). An average mouse weight of 25 g was used for the calculations.

Experimental Determined Data			Min. Concentration Needed to Reach Sufficient Imaging Contrast: Target-to-Background >2		Success Criteria	Is BBB Pretargeting Feasible?
TCO/Ab	Dosing [mg/kg]	Brain Uptake [%ID/g]	*Ab_min_*	*Ab_traffic_*	*Ab_traffic_ − Ab_min_ =*	
6.8	2	1.1	1.6	2.6	1	Yes
3	2	1.1	3.6	2.6	−1	No *
3	5	0.7	3.6	4.2	0.6	Yes
12	1	1.1	0.9	1.3	0.4	Yes

Positive values indicate that BBB pretargeting is possible. * However, the ratio was in the same order of magnitude, which indicates that pretargeting beyond the BBB might nevertheless be feasible.

## Data Availability

Data is contend within the article and Supplementary Materials.

## Supplementary Materials

The following supporting information can be downloaded at: https://www.mdpi.com/article/10.3390/ph15101191/s1. References [69,70,71] are cited in Supplementary Materials.
