# Supplementary material for: Pretargeted Imaging beyond the Blood–Brain Barrier—Utopia or Feasible?
Source: Pharmaceuticals (Basel). 2022 Sep 27;15(10):1191. doi: 10.3390/ph15101191 (PMC9612205; doi:10.3390/ph15101191)
Supplement: Supplementary file 1 [file pharmaceuticals-15-01191-s001.zip › pharmaceuticals-1842175-supplementary.pdf]

Supporting Information

Table of Contents

Section S1: Modification of antibodies with TCO-PEG$_4$-NHS ester 2
General procedure 2
Section S2: Quantification of TCO-loading on the antibodies 2
Section S3: TfR–Aβ ELISA 2
Section S4: Radiochemistry 3
Radiolabeling of Tz [^{18}F]**1** 3
Radiolabeling of of Tz [^{111}In]**2** 3
Section S5: Pretargeted autoradiography 3
Section S6: Determination of TCO-Ab bound to tissue 5
Section S7: Ex vivo biodistribution of [^{18}F]3D6scFv8D3 5
Animals 5
Animal injection and ex vivo biodistribution procedure 5
Section S8: TCO Stability in TCO-3D6scFv8D3 at different pH 6
Section S9: ELISA TfR 6
Section S10: Pretargeted Autoradiography Images 7
Section S11: Ex vivo biodistribution of [^{18}F]3D6scFv8D3 in blood and peripheral organs 7
References 8

Section S1: Modification of antibodies with TCO-PEG$_4$-NHS ester

General procedure

mAb 3D6 and mAb3D6scFv8D3 were provided by Uppsala University.[1] Lysine residues in mAb 3D6 and mAb3D6scFv8D3 were functionalized with TCO-PEG$_4$-NHS ester. Sodium carbonate buffer (1 M, pH 8.0) was added to PBS containing mAb 3D6 (0.1-1.0 mg/mL) to give a final concentration of 30 mM, followed by addition of equatorial TCO-PEG$_4$-NHS (Broadpharm, BP-22418) in acetonitrile using 10–1000 eq. TCO–antibody ligand molar ratio. The mixture incubated at 600 rpm for 2 hours at room temperature in the dark. The solution was purified to remove the unreacted TCO-PEG$_4$-NHS ester with Zeba spin desalting columns (7K MWCO, 0.5 mL, 89882, Thermofisher) and eluted in PBS (pH 7.4). Final protein concentration was measured with Nanodrop (NanoDrop 2000, ThermoScientific).

Section S2: Quantification of TCO-loading on the antibodies

Titration experiments were conducted to quantify the amount of reactive TCOs per TCO-mAb. Aliquots of TCO-mAb in PBS (5 µL of 0.1-1.0 mg/mL solutions) were mixed with aliquots of ^{111}In-labeled Tz ([^{111}In]**2)** stock (5 µL, Section S4) containing 2-3 eq. of Tz per expected amount of TCOs on the TCO-mAbs, and the mixed samples were incubated at 600 rpm for 1 hour at 37 °C. NuPAGE™ LDS Sample Buffer (3 µL, NP0007, Invitrogen) was added to each sample and the samples were heated to 70 °C and shaken vigorously for 10 minutes. Afterwards, samples were applied onto SDS-PAGE gels (NuPAGE™ 4 to 12%, Bis-Tris, 1.0 mm, Mini Protein Gel, 12-well, NP0322BOX , Invitrogen). SDS-PAGE was run in MES-SDS buffer at 150V for 40-45 min. Separation was monitored by running SeeBlue® Plus2 Pre-Stained Protein Standard along with ^{111}In-Tz samples. Control mixtures of 5 µL of ^{111}In-labeled Tz solution with 15 µL of PBS were prepared and processed in the same manner. SDS-PAGE gels were exposed to phosphor storage screens and read by a Cyclone Storage Phosphor System (PerkinElmer Inc.). Quantification of plate readings was done with Optiquant software (version 3.00, Packard Instruments Co). Radioactivity found in the section of the gel corresponding to molecular weights of 38 kDa and above (using SeeBlue standards as reference) was considered mAb-bound. The percentage of the total mAb-bound activity per lane of the SDS-PAGE gel was used to calculate the number of reactive TCOs per mAb using the formula below.

$$N_{TCO} = \frac{\%bound \times n_{Tz}}{RCP_{Tz} \times n_{mAb}},$$

Where N_{TCO} is the number of reactive TCOs per mAb;

%bound is the percentage of mAb-bound activity;

n_{Tz} is the amount of ^{111}In-labeled Tz in the aliquot;

n_{mAb} is the amount of TCO-mAb in the aliquot;

RCP_{Tz} is the radiochemical purity of ^{111}In-labeled Tz determined by radio-HPLC.

Section S3: TfR–Aβ ELISA

TCO-3D6scFv8D3 and TCO-3D6 were tested with ELISA for retained binding to TfR and Aβ, respectively. The 96-well plates (Corning Inc.) were coated at 4 °C overnight with 1 µL/mL, 50 µL well murine TfR (1 µL/mL, 50 µL well, Nordic BioSite, 158-50741-M07H-50) or 50 nM, 50 µL well Aβ (SP-BA40-1 Innovagen). After 1 h blocking with 1% BSA in PBS, a serial dilution of TCO-mAb was added to the TfR or Aβ protofibril-coated wells and incubated for 1.5 h on a shaker at room temperature and detected by a 1-h incubation with HRP-

conjugated 1:2000 diluted HRP-conjugated anti-mouse-IgG (Invitrogen 616520). Signals were developed with K blue aqueous TMB substrate (Fischer Scientific, 11849260) and read with a spectrophotometer at 450 nm. All antibody dilutions were made in ELISA incubation buffer (PBS with 0.1% BSA, 0.05% Tween and 0.15% Kathon).

Section S4: Radiochemistry

Radiolabeling of Tz [^{18}F]**1**

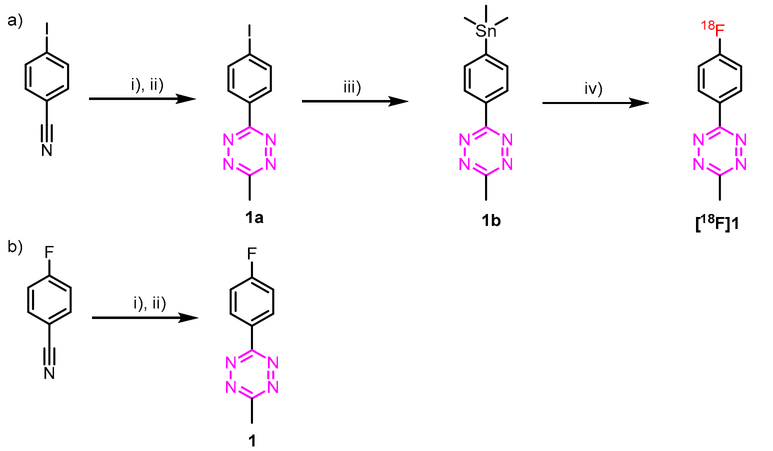

Scheme S1: *Schematic overview to synthesize Tz precursors (2),, Tz reference compounds (3) and radiolabeled ^{18}F-Tz ([^{18}F]3). i) MeCN, Zn(OTf)2, NH2NH2 . H2O, EtOH, 60 ºC, 24 h; ii) NaNO2, AcOH, 0 ºC, 20 min, 39% (1), 20% (4), 30% (6); iii) (Me3Sn)2, Pd(OAc)2, meCgPPh, THF, 70 ºC, MW, 30 min, 76%; iv) B2pin2, Pd2dba3, XPhos, KOAc, 1,4-Dioxane, 24 h, 110ºC, 63%; v) Cu(OTf)2, pyridine, [^{18}F]KF, DMA, 5 min, 100 °C.*

Radiolabeling of of Tz [^{111}In]**2**

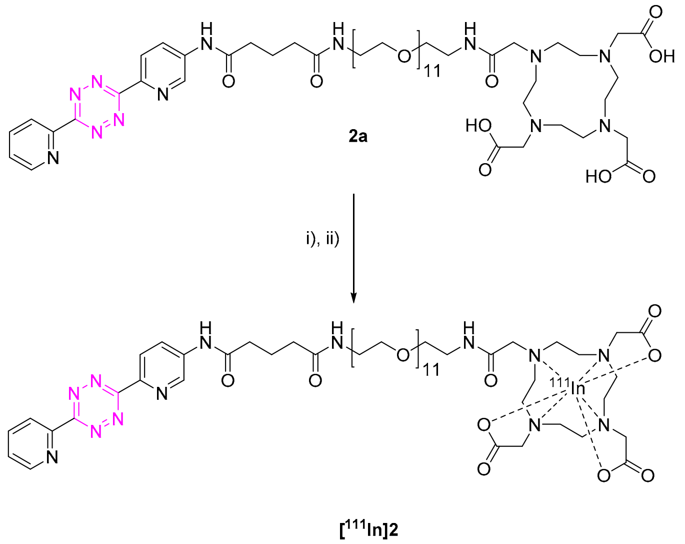

Scheme S3. Preparation of [^{111}In]3. i) ^{111}InCl, 1M NH4OAc, 60 ºC, 5 min ii) 10 mM DTPA, gentisic acid, 60 ºC, 5 min

Section S5: Pretargeted autoradiography

Pretargeted autoradiography was performed according to previously published procedure.[2] A schematic overview is provided in Figure S1.

Frozen brains of old (~18 months of age; 25-40 g body weight) male C57BL/6 mice and tg-ArcSwe model harboring the Arctic (AβPP E693G) and Swedish (AβPP KM670/671NL) mutations (provided by Uppsala University) were cut into halves along the sagittal symmetry plane.[3] Each half was mounted, lateral side up, on the cryostat sample holder pretreated by Tissue-Tek OCT Compound, and fixed by freezing in the cryostat. After fixing, brains were cut at −22 °C into sagittal slices 20 µm thick using a Leica microtome, and the slices were thaw-mounted on Superfrost (70 × 22 mm, Fischer) adhesive slides. Only slices containing both cortex (specific binding) and cerebellar regions (representing nonspecific binding) were used. The slices were allowed to dry and were then put into storage boxes and kept at −80 °C until they were used.

On the day of the experiment, the frozen slides with mounted brain sections were allowed to come to room temperature for 30 min. Afterwards, a PAP pen was applied around the sections and 600 µL 1% BSA in PBS (pH 7.4) with 0.05% Tween 20 was added and incubated at room temperature for 30 minutes in incubation boxes. Afterwards, the BSA solution was removed and 600 µL of PBS (pH 7.4) with 0.05% Tween 20 was applied and incubated at room temperature for 5 minutes. The preincubation buffer was removed and 600 µL of TCO-3D6 or TCO-3D6scFv8D3 (0.06 µL/mL and 0.006 µL/mL) in PBS (pH 7.4) with 0.05% Tween 20 was applied and incubated overnight at room temperature in a moist incubation box. On the second day, the TCO-mAb solution was removed and the slides were washed in cold PBS (pH 7.4) (3x15 min) and cold demineralized water (1x30 sec). Then, 600 µL 20 nM [^{111}In]**2** solution was applied to all sections and incubated for 60 minutes. Afterwards, the radioactive solution was removed and the slides were washed in cold PBS (pH 7.4) (3x5 min) and demineralized water (1x30 sec) and dried by a flow of compressed air. A calibration curve with dilutions of [^{111}In]**2** - 40nM, 20nM, 6.7nM, 2.2nM, 0.74nM, 0.25nM and 0.12nM – was prepared. After drying the plates, the slides were exposed on phosphor storage screens for 1-12 hours, depending on the amount of radioactivity on the slide. Afterwards, the phosphor storage screens were read by a Cyclone Storage Phosphor System (Packard Instruments Co). Quantification of plate readings was done with Optiquant software (version 3.00, Packard Instruments Co) and ImageJ by drawing regions of interest on the cortex and cerebellum manually. Data was processed in Excel and cortex/cerebellum imaging contrast ratios were calculated.[4,5]

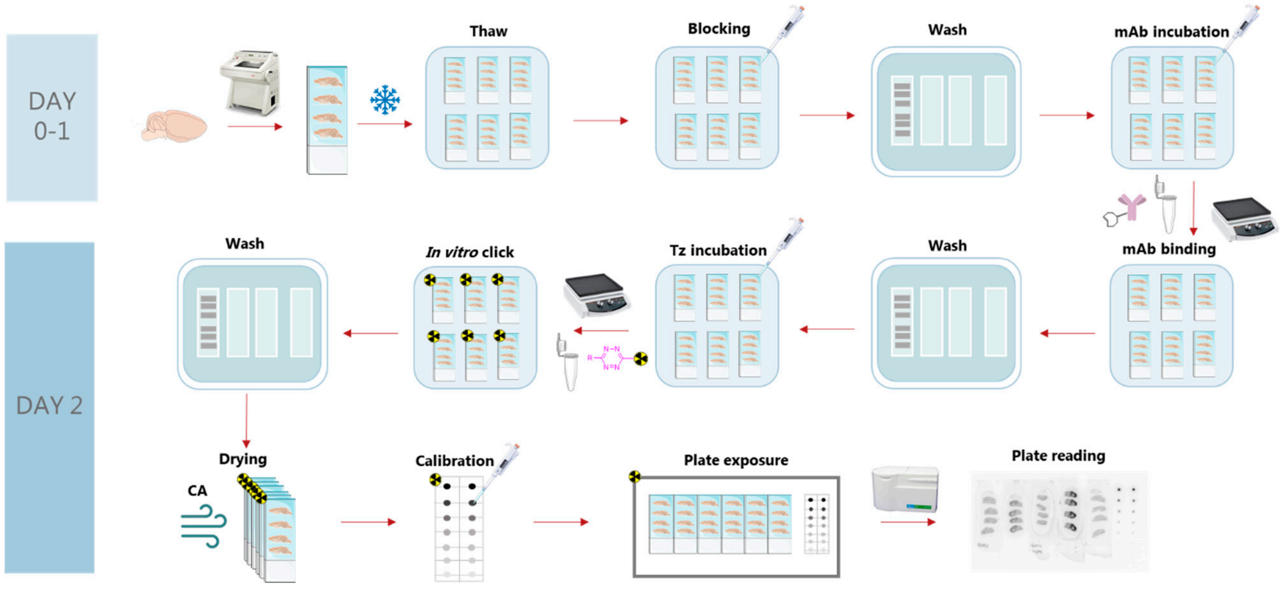

Figure S1: Schematic overview of the pretargeted autoradiography protocol.

Section S6: Determination of TCO-Ab bound to tissue

Slides with brain sections from tg-ArcSwe mice were prepared as described in Section S6. Frozen brain slides were allowed to come to room temperature for 30 min. A PAP pen was applied around the sections and 600 µL 1% BSA in PBS (pH 7.4) with 0.05% Tween 20 was added and incubated at room temperature for 30 minutes in incubation boxes. Afterwards, the BSA solution was removed and 600 µL of PBS (pH 7.4) with 0.05% Tween 20 was applied and incubated at room temperature for 5 minutes. The preincubation buffer was removed and 600 µL of [^{111}In]3D6scFv8D3 (0.06 µL/mL and 0.006 µL/mL) in PBS (pH 7.4) with 0.05% Tween 20 was applied and incubated overnight at room temperature in a moist incubation box. [^{111}In]3D6scFv8D3 was prepared as described in Section S4. On the second day, the [^{111}In]3D6scFv8D3 solution was removed and the slides were washed in cold PBS (pH 7.4) (3x5 min) and cold demineralized water (1x30 sec). After drying the plates, the brain tissue was wiped of with tissue paper and placed in γ-counting tubes and measured by counted by γ-counter Cobra II Auto-Gamma Counter (Model D5005, Packard BioScience Company). Quantification of the γ-counts was conducted in excel to determine the TCO-Abs bound to the tissue.

Section S7: Ex vivo biodistribution of [^{18}F]3D6scFv8D3

Animals

Ex vivo distribution experiments were performed with 12-week old male C57BL/6JRj mice purchased from Janvier Labs. Mice were maintained at Panum, AEM Unit 16.2, University of Copenhagen and arrived 4 weeks prior to the experiments for habituation in the new environment. Mice were housed together in individually ventilated cages in a specific pathogen-free, humidity and temperature-controlled facility (12h light/dark cycle) with free access to water and chow. All procedures were conducted following the European Commission's Directive 2010/63/EU, FELASA and ARRIVE guidelines for animal research and, with approval from The Danish Council for Animal Ethics (license number 2020-15-0201-00581).

Animal injection and ex vivo biodistribution procedure

For the ex vivo biodistribution of the [^{18}F]3D6scFv8D3, mice (n=3) were anaesthetized with the injectable anesthesia mix of ketamine (100mg/kg) and dexmedetomidine (0,5mg/kg) and kept in anesthesia during the whole experiment. To maintain a physiological body temperature, mice were kept on a warming pad. All animals were tested for pain response by toe pinching. 26G venous catheters (26G BD Neoflon™, VWR) were inserted in the lateral tail veins and fixed with tape. 50 µg of ^{18}F-3D6scFv8D3 (equaling a volume of 120ul and between 0.5 to 0.7 MBq per mouse) were injected followed by a saline flush of 80 µl. 2 hours after antibody injection, mice were euthanized by an injection of a lethal dose of ketamine/dexmedetomidine (300mg/kg & 1.5mg/kg). Blood was collected via cardiac puncture and separated into a plasma and a blood cell fraction using EDTA tubes centrifuged at 4°C at 2000xg for 10 minues.

After blood collection, mice were perfused with ice-cold saline and the following tissues were collected: heart, lungs, bladder, liver, stomach, gall bladder, kidneys, bone, muscle, scull, spleen, right brain hemisphere, left cerebellum and left cerebrum. All tissues were wet-weighed and counted on an automated γ-counter Cobra II Auto-Gamma Counter (Model D5005, Packard BioScience Company). The concentration of radioactivity in blood and tissues was determined as percentage injected dose per gram (%ID/g). Data were expressed as mean±SD (*n*=3).

Section S8: TCO Stability in TCO-3D6scFv8D3 at different pH

TCO-3D6scFv8D3 containing approx. 8 TCOs/Ab in PBS was divided into three aliquots and the pH was altered to give a final pH of 4, 5, and 7.4 respectively. After 0, 2, 4, 24, 48 and 72 hours, an aliquot of each vial was taken and an excess of ^{111}In-Tz (1.5 eq) was added. The mixtures were incubated for one hour at 37˚C and measured by radioHPLC (Aeris™ 3.6 µm Widepore C4 200 Å, LC Column 150 x 4.6 mm, Solvent A = 0.1% TFA in water, solvent B = 0.1% TFA in acetonitrile. HPLC elution method: 0-4.5 min – 25% B, 4.5-8 min - gradient from 25% B to 90% B, 8-10.5 min – 90% B, 10-12.5 min - back to 25% B, 12.5-15 min – 25% B; flow rate 1.5 mL/min). Peaks were integrated to determine the RCC.

Section S9: ELISA TfR

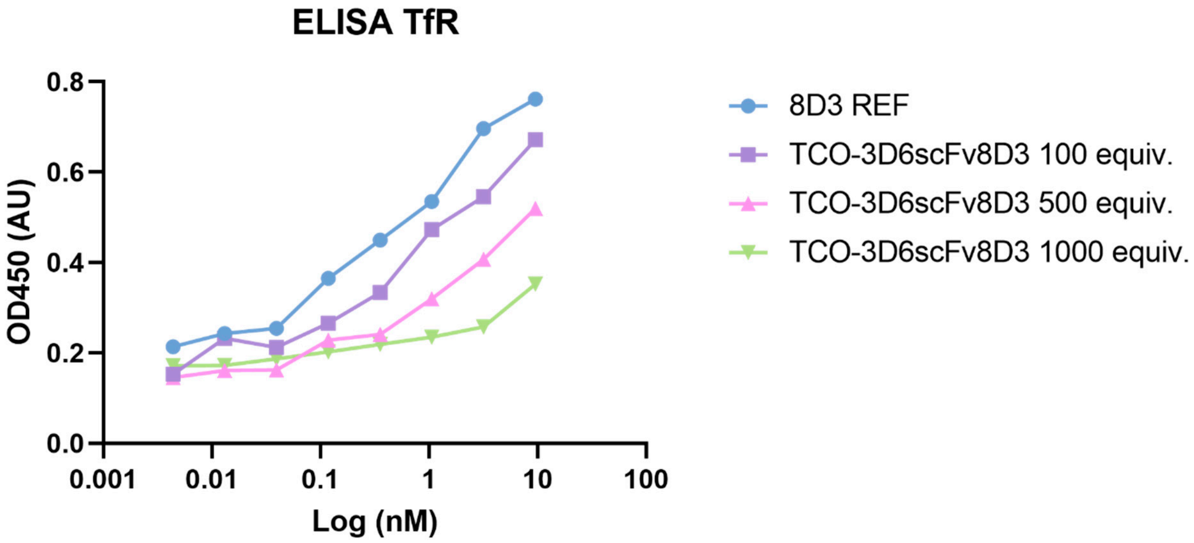

Figure S2: TfR ELISA data of TCO-3D6scFv8D3 conjuates with different TCO-PEG4-NHS equiv: 100 equiv. (approx. 6 TCO/Ab), 500 equiv. (approx. 18 TCO/Ab) and 1000 equiv. (approx. 19 TCO/Ab). 8D3 REF is unmodified 3D6scFv8D3.

Section S10: Pretargeted Autoradiography Images

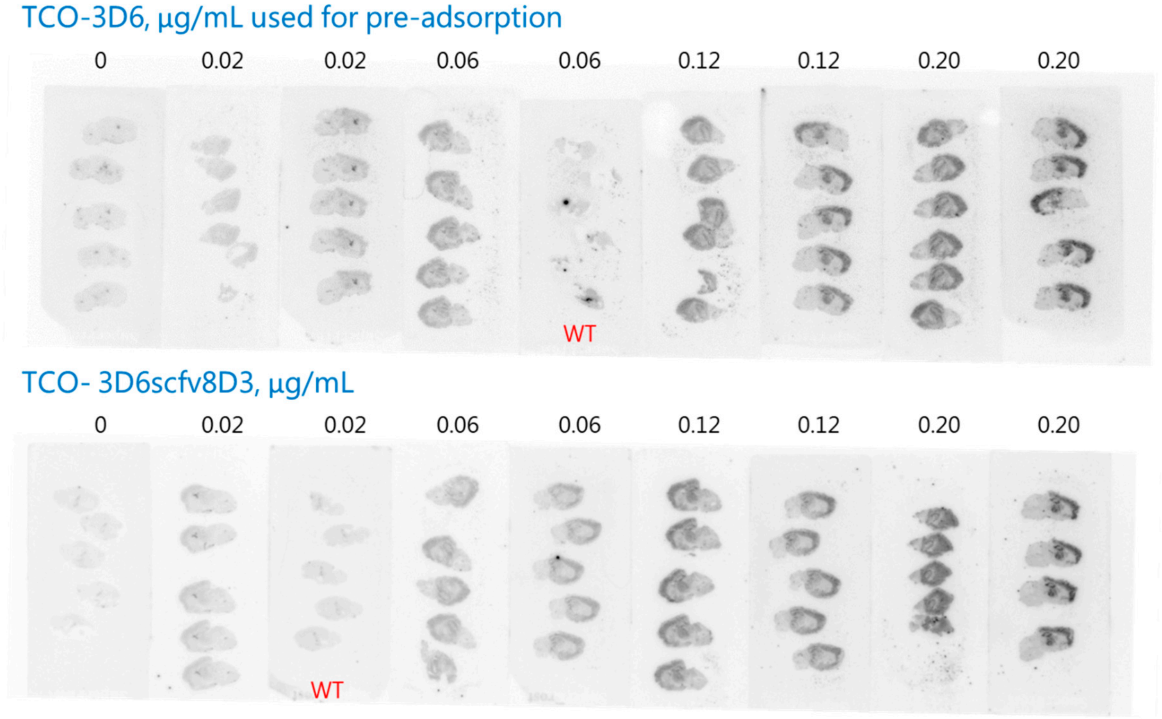

Figure S3: Pretargeted autoradiography images with TCO-3D6 and TCO-3D6scFv8D3 using different concentrations of TCO-Ab.

Section S11: Ex vivo biodistribution of [^{18}F]3D6scFv8D3 in blood and peripheral organs

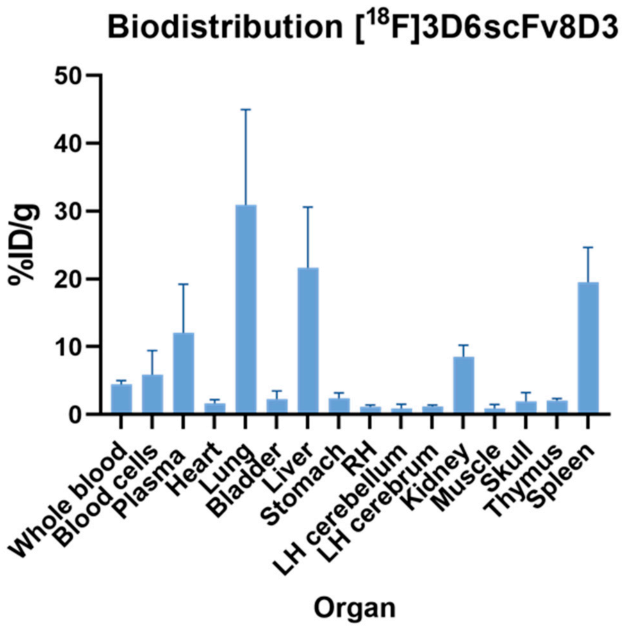

Figure S4: Biodistribution data of [^{18}F]3D6scFv8D3 two hours post injection (n=3).. RH = Right hemisphere, LH = left hemisphere. Main uptake is observed in the lungs, liver, spleen and kidneys.

References

1. Fang, X. T. *et al.* High detection sensitivity with antibody-based PET radioligand for amyloid beta in brain. *Neuroimage* **184**, 881–888 (2019).

2. Shalgunov, V. *et al.* Pretargeted Imaging Beyond the Blood-Brain Barrier. *ChemRxiv* **Cambridge**, (2022).

3. Meyer, P. T., Elmenhorst, D., Zilles, K. & Bauer, A. Simplified quantification of cerebral A1 adenosine receptors using [18F]CPFPX and PET: Analyses based on venous blood sampling. *Synapse* **55**, 212–223 (2005).

4. Raval, N. R. *et al.* P.804A single dose of psilocybin increases synaptic density and decreases 5-HT2A receptor density in the pig brain. *European Neuropsychopharmacology* **40**, S453–S454 (2020).

5. Griem-Krey, N., Klein, A. B., Herth, M. & Wellendorph, P. Autoradiography as a Simple and Powerful Method for Visualization and Characterization of Pharmacological Targets. *Journal of Visualized Experiments* (2019) doi:10.3791/58879.
